# Leucine Enhances Stress Resistance in Honeybees (*Apis mellifera* L.) by Modulating *Sestrin*-Dependent Antioxidant Responses

**DOI:** 10.3390/biology15141124

**Published:** 2026-07-10

**Authors:** Zhe Wang, Zhenguo Liu, Ge Zhang, Hongfang Wang, Xuepeng Chi, Ying Wang, Baohua Xu

**Affiliations:** 1 Department of Animal Science, Shandong Agricultural University, Tai’an 271017, China; wangz3006@163.com (Z.W.); lzhenguo_001@163.com (Z.L.); gezhang.bee@gmail.com (G.Z.); wanghongfang22@163.com (H.W.); xixuepeng@126.com (X.C.); wangying@sdau.edu.cn (Y.W.); 2 Key Laboratory of Efficient Utilization of Non-Grain Feed Resources (Co-Construction by Ministry and Province), Shandong Agricultural University, Tai’an 271017, China; 3 Shandong Provincial Key Laboratory of Animal Nutrition and Efficient Feeding, Tai’an 271017, China

**Keywords:** leucine, *sestrin*, antioxidant defense, *Apis mellifera*

## Abstract

Environmental stressors such as heavy metals and pesticides pose significant threats to honeybee health by inducing oxidative stress. Enhancing nutritional supply has emerged as a potential strategy for honeybees to enhance stress resistance. However, the underlying molecular mechanisms remain poorly understood. This study investigated the role of leucine (Leu) in regulating antioxidant responses in honeybees (*Apis mellifera* L.), with a focus on the involvement of *sestrin* and the Target of Rapamycin (TOR) signaling pathway. The results showed that Leu supplementation activated the TOR signaling pathway and upregulated *sestrin* expression, leading to enhanced antioxidant capacity and reduced reactive oxygen species (ROS) levels. Silencing *sestrin* disrupted antioxidant homeostasis, increased stress susceptibility, and caused midgut damage, confirming its essential role in oxidative stress resistance. Notably, inhibition of TOR did not prohibit the protective effects of Leu, suggesting that *sestrin*, rather than TOR, serves as a key mediator. Furthermore, Leu fine-tuned antioxidant responses under different stress conditions through *sestrin*. Overall, this study demonstrates that Leu and *sestrin* play a central role in maintaining antioxidant homeostasis and provide a theoretical basis for nutritional strategies to improve honeybee health and stress resilience.

## Simple Summary

Honeybees are essential for crop pollination and ecological balance, yet their health is increasingly compromised by environmental stressors such as heavy metals and pesticides. These stressors induce oxidative stress, a cellular imbalance that damages tissues and impairs bee survival. This study investigated whether leucine, a common amino acid in pollen, can enhance the natural antioxidant defenses of honeybees. Our experiments showed that supplementing bees with leucine significantly increased their capacity to counteract oxidative stress, reduced cellular damage, and improved survival under stress conditions. Further analysis revealed that leucine acts by activating *sestrin*, which serves as a key regulator of antioxidant responses. Notably, the protective effect of leucine did not primarily depend on the previously recognized TOR pathway. When *sestrin* was experimentally silenced, bees became highly susceptible to stress and developed gut damage, confirming its essential role. Importantly, leucine partially restored resilience even when *sestrin* was absent, suggesting additional protective mechanisms. These findings demonstrated that dietary leucine supplementation offers a feasible nutritional strategy to strengthen honeybee health and stress resistance.

## 1. Introduction

The global population of honeybees (*Apis mellifera* L.) is widely threatened by a range of environmental stressors, including heavy metal contamination, pesticide exposure, and climate change [1,2]. These stressors can induce oxidative stress, which is characterized by excessive production of free radicals, overloading the antioxidant capacity of organisms and leading to cellular damage and functional impairment [3,4]. Among these factors, heavy metal pollution has become a growing global concern [5]. Although cadmium (Cd) concentrations in agricultural soils are typically below 10 μg/L [6,7], Cd can be readily transferred from soil to crops, where it accumulates and enters the food chain, thereby posing potential risks to both animal and human health [8]. In honeybees, sublethal doses of Cd have been shown to impair olfactory learning performance, accompanied by disrupted expression of genes involved in oxidative stress responses and odor perception in the brain [9]. In addition to heavy metals, pyrethroid insecticides are widely used as effective alternatives to organophosphates because of their high insecticidal activity and relatively low toxicity to mammals [10,11]. However, their widespread application may still exert adverse effects on non-target beneficial organisms, particularly pollinators [12].

Despite these environmental challenges, adequate intake of high-quality pollen has been shown to significantly enhance disease resistance, strengthen immune responses, and alleviate the adverse effects of various stressors in honeybees [13,14]. As the primary source of protein nutrition for bees, pollen provides essential amino acids required for growth, development, and physiological homeostasis [15]. Among these, leucine (Leu) is particularly abundant and plays a critical role in organismal development, acting as an effective regulator of protein synthesis and being sufficient on its own to activate the Target of Rapamycin (TOR) signaling pathway [16]. Under stress conditions, such as energy depletion, reactive oxygen species accumulation, or DNA damage, TOR activity is typically inhibited [17]. Sestrins are evolutionarily conserved proteins that accumulate under stress and function as key sensors regulating TOR signaling [18,19].

Accumulating evidence indicates that sestrins serve as key mediators linking nutritional signaling and oxidative stress responses [20]. Their expression is induced by a variety of stress stimuli, enabling them to regulate fundamental cellular processes such as cell survival, antioxidant defense, growth, and metabolism [21,22]. In vitro studies have further shown that sestrins possess oxidoreductase activity, suggesting they may function directly as antioxidant proteins [23]. Collectively, these findings indicate that sestrins modulate redox homeostasis through the mTOR and other pathways, which have been observed in various model organisms including slime molds, worms, fruit flies, and mice [24].

Despite accumulating evidence that Leu activates TOR signaling and that sestrin functions as a key regulator of nutrient sensing and antioxidant defense, whether Leu regulates *sestrin* expression in honeybees and how Leu, TOR, and *sestrin* coordinately mediate antioxidant responses under environmental stress remain unclear. Moreover, whether this regulatory network contributes to protection against heavy metal- and pesticide-induced oxidative damage remains unclear. Therefore, the present study investigated the role of Leu in enhancing antioxidant capacity in honeybees (*Apis mellifera* L.) using indoor rearing, RNA interference (RNAi), quantitative real-time PCR (qRT-PCR), and other experimental approaches. Specifically, we aimed to determine how Leu regulates TOR signaling and *sestrin* expression, and whether this regulatory network contributes to antioxidant defense against heavy metal and insecticide exposure. By elucidating the regulatory roles of Leu in TOR signaling and *sestrin* expression, this study not only provides a theoretical basis for developing nutritional intervention strategies to protect honeybee health, but also identifies *sestrin* as a potential molecular target for improving stress resistance in bees.

## 2. Materials and Methods

### 2.1. Honeybee Rearing

Honeybees (*Apis mellifera* L.) were obtained from the apiary of the College of Animal Science and Technology, Shandong Agricultural University (Tai’an, China) from May to July 2025. Five healthy sister colonies with similar colony conditions were selected. Newly emerged worker bees were collected from these colonies and raised in wooden cages (CN204047589U: 23 cm × 9.5 cm × 5 cm, length × width × height). Bees were kept in an incubator at constant temperature (33 °C) and relative humidity (approximately 50%) and fed with 50% sucrose solution. Leu was added to the sucrose solution at a concentration of 1.5 mg/g, which was identified as the optimal dose through preliminary experiments.

Two hundred new workers were captured and randomly divided into 2 groups. The control group (CK) was fed with 50% sugar solution, and the Leu group was fed with 50% sugar solution supplemented with 1.5 mg/g Leu (Hebei Huayang Biotechnology Co., Ltd., Jingmen, China). Thirty bees in each group were exposed to CdCl_2_ (1 mg/L, Shanghai Macklin Biochemical Co.,Ltd., Shanghai, China; C805625, purity ≥ 99%) or permethrin (10 mg/L, ANPEL-TRACE Standard Technical Services Co. Ltd., Shanghai, China; D0003716, purity ≥ 97%) on the 8th day, and the numbers of dead bees were recorded daily. Other surviving bees were not subjected to heavy metal or pesticide treatments and were sampled on the 9th day.

Samples were collected from multiple developmental stages, including larvae (L1-L5), prepupae (PP), white-eyed pupae (Pw), pink-eyed pupae (Pp), brown-eyed pupae (Pb), newly emerged workers (NE), 9-day-old bees and 18-day-old bees. In addition, specific tissues were dissected from 9-day-old bees, including the head, thorax, fat body, wings and guts. All samples were immediately frozen in liquid nitrogen and stored at −80 °C for qRT-PCR.

### 2.2. qRT-PCR

Total RNA was extracted from bees using RNAex Pro Reagent (Accurate Biology Co., Ltd., Changsha, China). The cDNA was synthesized using Evo M-MLV RT Premix (Accurate Biology Co., Ltd., Changsha, China) according to the manufacturer’s instructions. The PCR process steps included 15 min at 37 °C and 5 s at 85 °C. qRT-PCR was performed with SYBR Green Pro Taq HS Premix (Accurate Biology Co., Ltd., Changsha, China). The PCR process steps included 30 s at 95 °C, 40 cycles of 5 s at 95 °C and 34 s at 60 °C. The primers were designed and synthesized by Sangon Biotech (Shanghai, China), and detailed information can be found in Table S1. The relative mRNA levels were calculated by the 2^−ΔΔCT^ method with *β-actin* as the reference gene [25]. All treatments were carried out in five independent biological replicates.

### 2.3. Determination of Antioxidant Enzyme Activity

The guts of the honeybees were dissected, and the remaining part was used to prepare 10% (*w*/*v*) homogenates for the determination of antioxidant enzyme activities and oxidative stress-related indicators. All treatments were carried out in five independent biological replicates, and each replicate consisted of two bees. The activities of superoxide dismutase (SOD), catalase (CAT), and total antioxidant capacity (T-AOC) in bees were measured according to the manufacturer’s instructions (Jiangsu Aidisheng Biological Technology Co., Ltd., Nanjing, China). The content of malondialdehyde (MDA) and hydrogen peroxide (H_2_O_2_) was measured according to the manufacturer’s instructions (Jiangsu Aidisheng Biological Technology Co., Ltd., Nanjing, China). The content of ROS was measured according to the manufacturer’s instructions (Shanghai Enzyme-linked Biotechnology Co., Ltd., Shanggai, China). All measurements were normalized by total protein concentration, which was determined using a ProtQuanti BCA Protein Quantification Kit (Accurate Biology Co., Ltd., Changsha, China).

### 2.4. TOR Activity Inhibition

Rapamycin (Rapa, Shanghai Aladdin Biochemical Technology Co., Ltd., Shanghai, China; S293790, purity ≥ 98%) was dissolved in anhydrous ethanol to prepare a 2 mg/mL stock solution. Two hundred newly emerged bees were randomly assigned to three groups. The EtOH group was fed sucrose solution containing 1% (*v*/*v*) anhydrous ethanol. The Rapa group was fed sucrose solution supplemented with 1% (*v*/*v*) rapamycin stock solution, yielding a final rapamycin concentration of 0.02 mg/mL. The Rapa + Leu group received the same solution as the Rapa group but with an additional 1.5 mg/g Leu. No treatment was applied during the first 4 days. From the 5th day, bees were provided with sucrose solution containing rapamycin and Leu for free feeding. The experimental dosage was referenced from Patel et al. [26]. Thirty bees in each group were treated with CdCl_2_ (1 mg/L) or permethrin (10 mg/L) on the 8th day, and bee mortality was recorded daily. The remaining bees were not treated, and surviving bees were sampled on the 9th day. The midguts were dissected, and the remaining bodies were used to measure immune enzyme levels. Gene expression was analyzed using whole-bee samples.

### 2.5. Bioinformatic Analysis

The sestrin amino acid sequence was obtained from NCBI (https://www.ncbi.nlm.nih.gov/, accessed on 20 May 2025). Amino acid sequences were aligned using DNAMAN v6.0 software, and a phylogenetic tree was constructed using the neighbor-joining method by the MEGA 11 software. Sestrin from *Apis mellifera* L. was used as a template for sestrin homology modeling using the SWISS servers (https://swissmodel.expasy.org/, accessed on 14 June 2025). The three-dimensional structure of Leu was obtained from PubChem (PubChem CID: 6106). AutoDock v4.2 software (The Scripps Research Institute, La Jolla, CA, USA) was utilized to conduct the molecular docking between the sestrin protein and Leu. The figures were visualized by the PyMOL v2.5.0 software (Schrödinger, LLC, New York, NY, USA).

### 2.6. RNA Interference (RNAi)

Double-stranded RNA (dsRNA) was synthesized using a T7 High Efficiency Transcription Kit (TransGen Biotech, Beijing, China) according to the manufacturer’s instructions. Then, green fluorescent protein (GFP, XM_006558668.3) was selected as the control. Primer sequences are listed in Table S1. Bees were fed 5 µg/bee dssestrin (dsses) or dsGFP, respectively. After 24 h, 48 h and 72 h, bees were collected to evaluate the silencing efficiency of *sestrin* by qRT-PCR.

Three hundred newly emerged bees were randomly assigned to three groups. The control group (dsGFP) was fed 50% sucrose solution and administered 5 µg/bee dsGFP every two days. The dsses group was fed 50% sucrose solution and administered 5 µg/bee dssestrin every two days. The dsses + Leu group was fed with 50% sucrose solution containing 1.5 mg/g Leu and administered 5 µg/bee dssestrin every two days. Bees in each group were subjected to stress-free treatment (Con), CdCl_2_ (1 mg/L), or permethrin (10 mg/L) treatment on the 8th day, and bee mortality was recorded daily. Bees were exposed to CdCl_2_ or permethrin from the 8th day, and all surviving bees (including untreated controls) were collected on the 9th day for further analysis. Their midguts were dissected and soaked in 4% paraformaldehyde (G1101, Servicebio, Wuhan, China) for morphological analysis, while the remaining body tissues were used for the determination of immune enzyme activities. Gene expression was analyzed using whole-bee samples.

### 2.7. Histopathological Analysis

Midgut samples were soaked in polyformaldehyde for 24 h. Subsequently, they were rinsed with water, dehydrated through ethanol, embedded in paraffin, sectioned, and stained with hematoxylin and eosin (H&E) following the method described by P. Maiolino et al. [27]. Finally, the sections were mounted with neutral resin. The completed tissue sections were observed and photographed under an optical microscope (ECLIPSE 80i, Nikon, Tokyo, Japan).

For quantitative assessment of histological damage, midgut sections were scored semi-quantitatively based on two parameters: (1) epithelial integrity (0 = intact epithelium; 3 = moderate disruption; 5 = severe disruption with loss of epithelial continuity); (2) cellular vacuolization (0 = none; 3 = mild; 5 = severe). Histological damage score = sum of the above two scores (range 0–10). Five independent biological replicates were used for each treatment.

### 2.8. Statistical Analysis

The results are reported as means ± standard error (means ± SEM). Data visualizations were used GraphPad Prism (Version 9.5, GraphPad, La Jolla, CA, USA). Significant differences were determined using SPSS (IBM SPSS Statistics 26.0 software, Chicago, IL, USA). Normality of data distribution and homogeneity of variance were assessed using the Shapiro–Wilk test and Levene’s test, respectively. For comparisons between two groups of normally distributed data, an unpaired two-tailed Student’s *t* test was applied. Differences among three or more groups were evaluated by one-way analysis of variance (ANOVA) followed by Tukey’s post hoc test for multiple comparisons. Survival analysis was conducted using the Kaplan–Meier method, and differences between groups were assessed by the log-rank test. A *p* < 0.05 was considered statistically significant.

## 3. Results

### 3.1. Leu Activated the TOR Signaling Pathway

**Figure 1:**
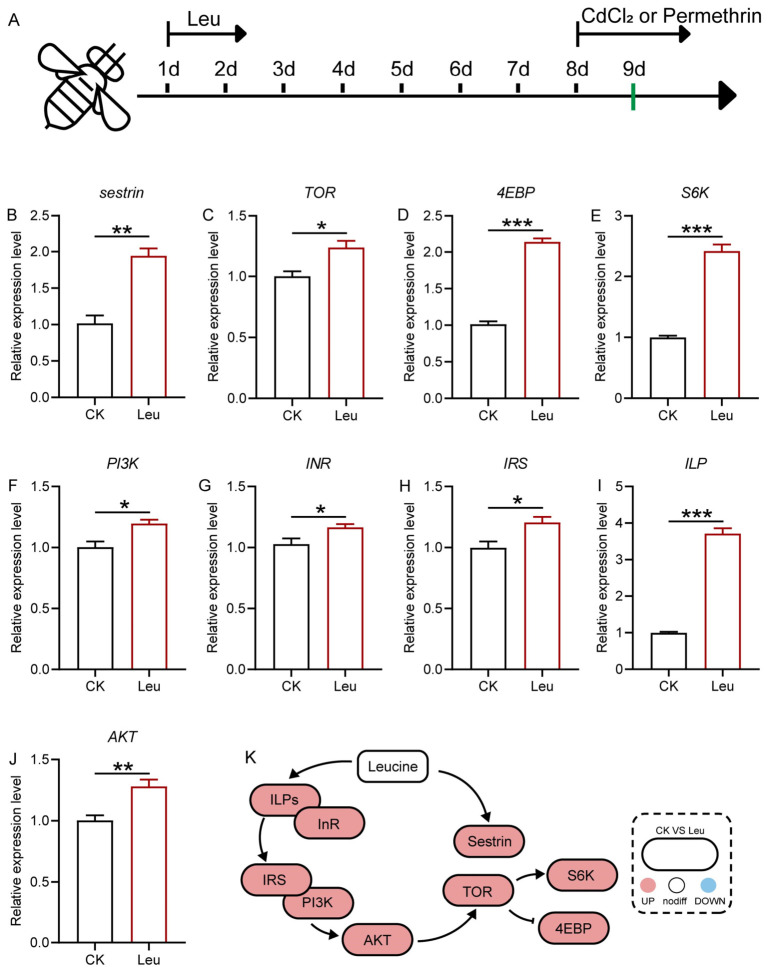
Leu affects the expression of the TOR signaling pathway. (**A**) Schematic of the feeding experiments: new bees were reared indoors. Honeybees in the CK group and groups with Leu were treated for 9 days. The mRNA expression levels of *sestrin* (**B**), *TOR* (**C**), *4EBP* (**D**), *S6K* (**E**), *PI3K* (**F**), *INR* (**G**), *IRS* (**H**), *ILP* (**I**) and *AKT* (**J**) after Leu treatment (*n* = 5). (**K**) Regulatory mechanism diagram of Leu on the TOR signaling pathway. The data were shown as the mean ± SEM. The significance of differences between two groups was calculated using a Student’s *t*-test. * *p* < 0.05, ** *p* < 0.01, *** *p* < 0.001.

To verify whether Leu can activate TOR signaling, we measured the expression of related genes in bees using qRT-PCR. The results indicated that Leu significantly increased the expression of *sestrin*, *TOR*, *4EBP*, *S6K*, *PI3K*, *INR*, *IRS*, *ILP*, and *AKT*, all of which are genes associated with the TOR signaling pathway (*p* < 0.05, Figure 1B–H).

### 3.2. Leu Alleviated Oxidative Stress in Bees

**Figure 2:**
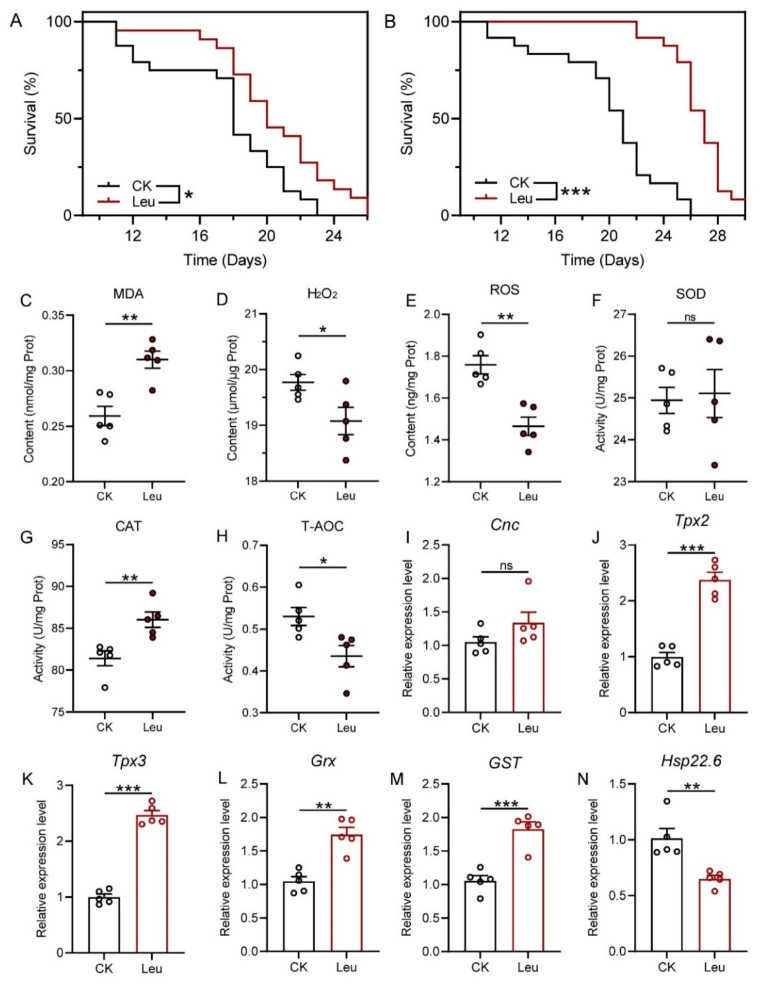
The effect of Leu on the antioxidant function of bees. The survival curve of bees exposed to CdCl_2_ (**A**) and permethrin (**B**) (*n* = 30). The activity of MDA (**C**), H_2_O_2_ (**D**), and ROS (**E**) after Leu treatment. The content of SOD (**F**), CAT(**G**), and T-AOC (**H**) after Leu treatment. The mRNA expression levels of *Cnc* (**I**), *Tpx2* (**J**), *Tpx3* (**K**), *Grx* (**L**), *GST* (**M**), and *Hsp22.6* (**N**) after Leu treatment (*n* = 5). The data were shown as the mean ± SEM. The significance of differences between two groups was calculated using a Student’s *t*-test. * *p* < 0.05, ** *p* < 0.01, *** *p* < 0.001.

After bees were subjected to CdCl_2_ or permethrin stress, the addition of 1.5 mg/g Leu significantly increased the survival rate of bees under CdCl_2_ or permethrin stress (*p* < 0.05, Figure 2A,B). Dietary Leu deficiency has been reported to induce excessive ROS production [28]. Leu can alter the expression of antioxidant genes in the liver of young grass carp and juvenile blunt snout bream, and enhance antioxidant enzyme activity and non-enzymatic antioxidant content [29,30]. To investigate the mechanism by which Leu improves antioxidant capacity in bees, we measured the levels of antioxidant genes, antioxidant enzymes, and antioxidant products. The results showed that after supplementation with Leu, MDA content increased, while H_2_O_2_ and ROS levels decreased in bees (*p* < 0.05, Figure 2C–E). Additionally, CAT activity increased, while T-AOC activity decreased (*p* < 0.05, Figure 2G,H). The expression levels of *Tpx2*, *Tpx3*, *Grx*, and *GST* were significantly upregulated (*p* < 0.05, Figure 2J–M), whereas the expression of *Hsp22.6* was downregulated (*p* < 0.05, Figure 2N).

### 3.3. Leu Regulated Sestrin Beyond TOR Signaling

**Figure 3:**
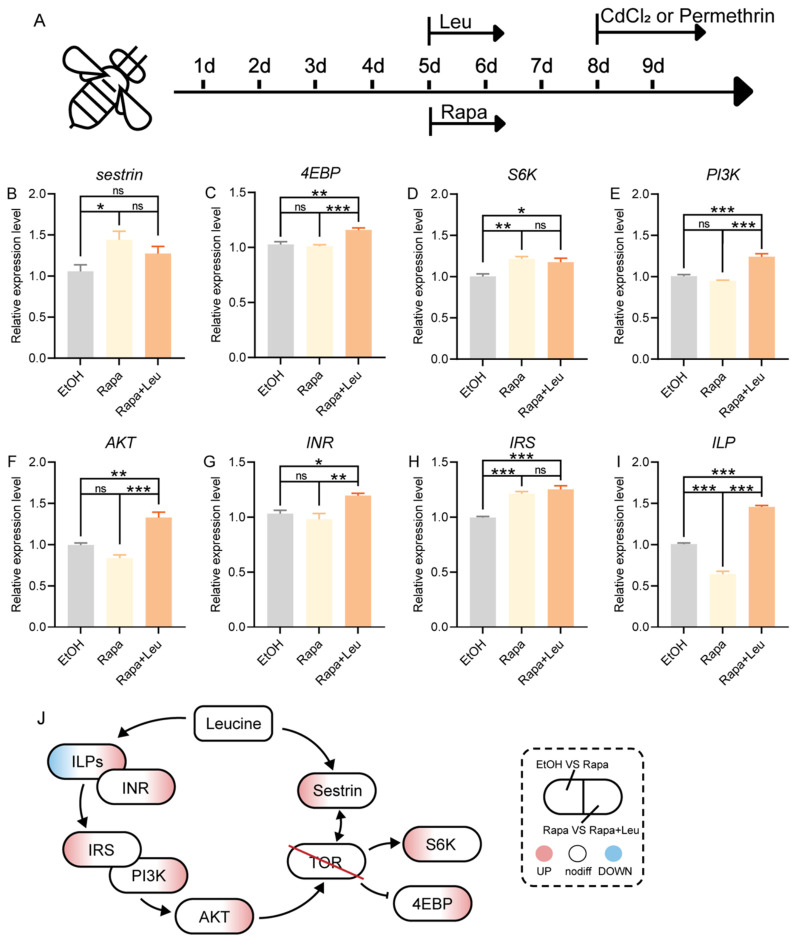
The effect of Leu on the TOR signaling pathway after treatment with Rapamycin. (**A**) Schematic of the feeding experiments: new bees were reared indoors and divided into the CK group, the Rapa group, and the Rapa + Leu group for 9 days. The mRNA expression levels of *sestrin* (**B**), *4EBP* (**C**), *S6K* (**D**), *PI3K* (**E**), *AKT* (**F**), *INR* (**G**), *IRS* (**H**), and *ILP* (**I**) after treatment (*n* = 5). (**J**) Regulatory mechanism diagram of Leu on the TOR signaling pathway with Rapamycin. The data were shown as the mean ± SEM. The significance of differences between three groups was calculated using one-way ANOVA followed by Tukey’s multiple comparison test. * *p* < 0.05, ** *p* < 0.01, *** *p* < 0.001.

To investigate whether Leu regulates *sestrin* expression via TOR, we inhibited TOR activity using rapamycin and measured the expression of related genes in 9-day-old bees by qRT-PCR. The results showed that after TOR inhibition, the expression levels of *sestrin*, *S6K*, and *IRS* were significantly increased, whereas supplementation with Leu restored these levels to those of the control group (*p* > 0.05, Figure 3B,D,H). The expression levels of *4EBP*, *PI3K*, *AKT*, and *INR* were not affected by TOR inhibition, but could be activated by Leu (*p* < 0.05, Figure 3C,E–G). The expression of *ILP* was influenced by both rapamycin and Leu (*p* < 0.05, Figure 3I).

### 3.4. TOR May Not Be the Key Factor in Alleviating Oxidative Stress in Bees

**Figure 4:**
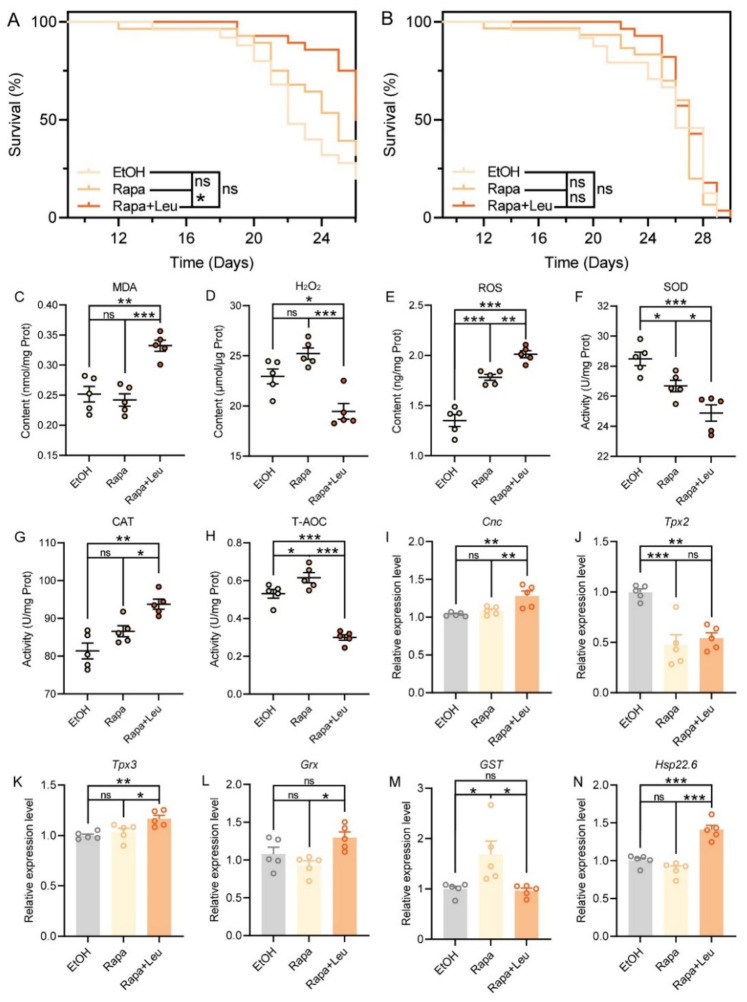
The effect of TOR on the antioxidant function of bees. The survival curve of bees exposed to CdCl_2_ (**A**) and permethrin (**B**) after Rapamycin treatment (*n* = 30). The activity of MDA (**C**), H_2_O_2_ (**D**), and ROS (**E**) after Rapamycin treatment. The content of SOD (**F**), CAT (**G**), and T-AOC (**H**) after Leu treatment. The mRNA expression levels of *Cnc* (**I**), *Tpx2* (**J**), *Tpx3* (**K**), *Grx* (**L**), *GST* (**M**), and *Hsp22.6* (**N**) Rapamycin treatment (*n* = 5). The significance of differences between the three groups was calculated using one-way ANOVA followed by Tukey’s multiple comparison test. * *p* < 0.05, ** *p* < 0.01, *** *p* < 0.001.

Previous studies have indicated that TOR is an important pathway for alleviating oxidative stress [31,32]. To investigate whether TOR serves as the key pathway in mitigating oxidative stress in bees, we assessed the survival rate of bees under stress conditions. The results showed that under CdCl_2_ or permethrin stress, inhibition of TOR activity had little effect on bee survival (*p* > 0.05, Figure 4A,B), whereas combined with Leu significantly increased the survival rate of bees exposed to CdCl_2_ (*p* < 0.05, Figure 4A). Further qRT-PCR analysis, enzyme activity assays, and antioxidant product measurements were performed to elucidate the mechanisms by which Leu and TOR influence antioxidant responses in bees. The results demonstrated that TOR activity did not affect CAT activity, MDA and H_2_O_2_ levels, or the expression of *Cnc*, *Tpx3*, *Grx*, and *Hsp22.6* (*p* > 0.05, Figure 4C,D,G,I,K,L,N). However, inhibition of TOR activity reduced the transcriptional level of *Tpx2* (*p* < 0.05, Figure 4J), while simultaneously increasing *GST* expression and T-AOC activity (*p* < 0.05, Figure 4G,M). Compared with the Rapa group, SOD and T-AOC activities decreased, and MDA and ROS levels increased in the Rapa + Leu group, and the expression levels of *Cnc*, *Tpx3*, *Grx*, and *Hsp22.6* were elevated (*p* < 0.05, Figure 4C,E–I,K,L,N).

### 3.5. Bioinformatics Analysis of Sestrin

**Figure 5:**
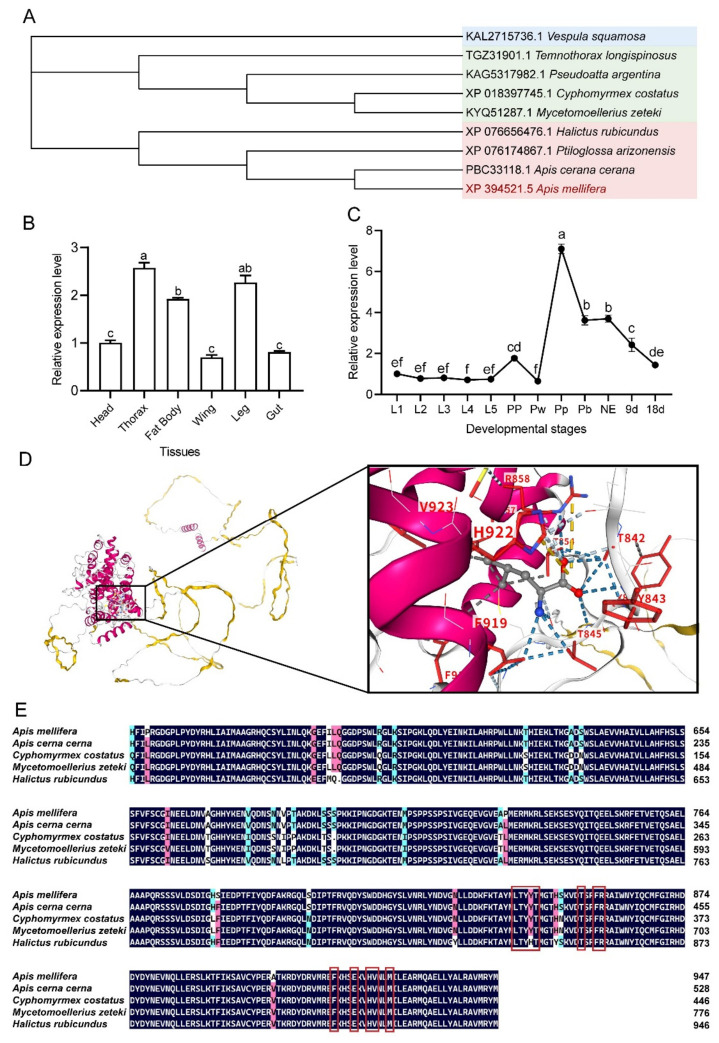
Characterization of sestrin in *Apis mellifera* and other species. (**A**) Phylogenetic tree created by the neighbor-joining method using MEGA 11 software. The sequences were obtained from the NCBI database. (**B**) Expression levels of *sestrin* in different parts of 9-day-old bees. The data were shown as mean ± SEM. Various letters above the bars indicate significant differences between groups (*p* < 0.05) as determined by one-way ANOVA followed by Tukey’s multiple comparison test. (**C**) Three-Dimensional diagram of the interaction between sestrin and Leu. (**D**) Multiple alignment analysis of sestrin from various species. The red box represents the predicted sestrin site connected to Leu. (**E**) Multiple sequence alignment of Sestrin orthologs from different species. The sequence data were retrieved from the NCBI database. Red boxes highlight the predicted binding sites between Leu and sestrin of *Apis mellifera*.

To predict the functional attributes of sestrin, a phylogenetic tree was constructed using MEGA 11 software based on the amino acid sequence of sestrin and its homologs from other species. The species included in the analysis are *Halictus rubicundus*, *Ptiloglossa arizonensis*, *Mycetomoellerius zeteki*, *Apis cerana*, *Cyphomyrmex costatus*, *Pseudoatta argentina*, *Vespula squamosa*, and *Temnothorax longispinosus*. The conserved and analogous amino acid sequences among these homologous proteins imply the potential functional similarity among them (78.90–97.36%). As shown in Figure 5A, sestrin from *Apis mellifera* L. is closely related to this protein from various insects, with the highest sequence similarity to *Apis cerana* (bootstrap value = 97.36%). The expression level of *sestrin* varied among different tissues, with the highest expression observed in the thorax, while lower expression levels were detected in the head, gut, and wings (*p* < 0.05, Figure 5B). On the other hand, *sestrin* expression did not change significantly during the larval stages (L1–L5), peaked at the Pp stage, and gradually decreased after the Pp stage (*p* < 0.05, Figure 5C). Molecular docking results showed that the binding free energy between Leu and sestrin was −5.2 kcal/mol (Center_x = −32, Center_y = 52, Center_z = 76; Size_x = 16, Size_y = 16, Size_z = 16). In addition, we predicted the binding sites between Leu and sestrin, and the predicted sestrin sites exhibited high conservation (Figure 5D,E).

### 3.6. Mutual Regulation Between Sestrin and TOR in Response to Leu

**Figure 6:**
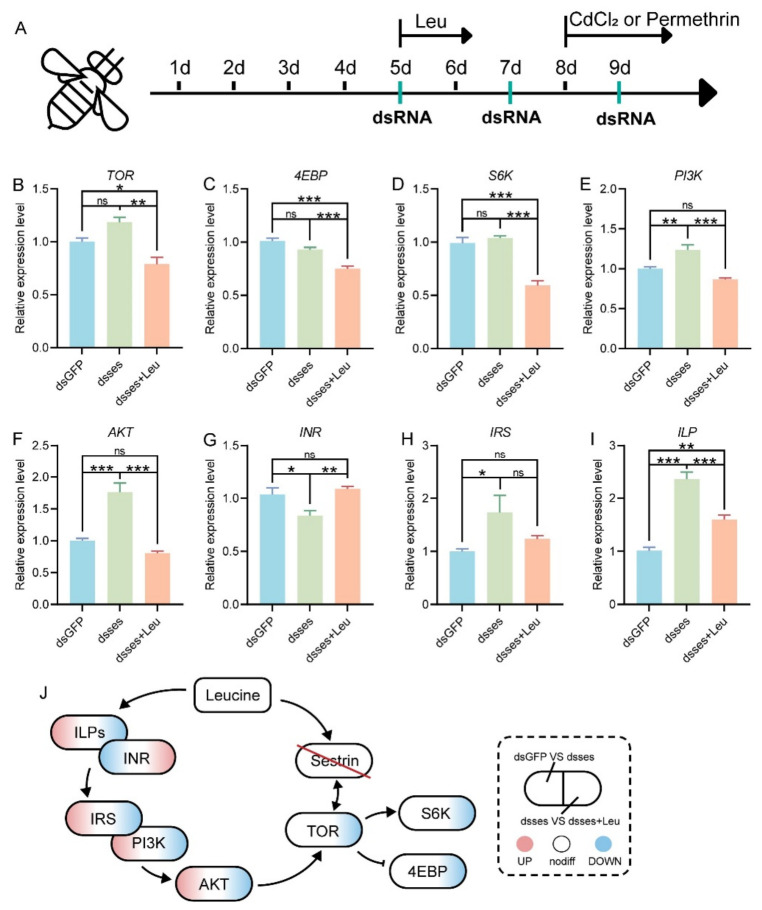
The effect of Leu on the TOR signaling pathway after treatment with dsses. (**A**) Schematic of the feeding experiments: new bees were reared indoors and divided into the CK group, the dsses group, and the dsses + Leu group for 9 days. The mRNA expression levels of *TOR* (**B**), *4EBP* (**C**), *S6K* (**D**), *PI3K* (**E**), *AKT* (**F**), *INR* (**G**), *IRS* (**H**), and *ILP* (**I**) after treatment (*n* = 5). (**J**) Regulatory mechanism diagram of Leu on the TOR signaling pathway with dsses. The significance of differences between the three groups was calculated using one-way ANOVA followed by Tukey’s multiple comparison test. * *p* < 0.05, ** *p* < 0.01, *** *p* < 0.001.

To explore how *sestrin* and TOR are regulated by Leu, the expression of TOR pathway-related genes was examined following *sestrin* silencing. As shown in Figure S1, administration of dssestrin significantly reduced the expression level of *sestrin*, and this suppression persisted for up to 48 h (*p* < 0.05). The results showed that following *sestrin* silencing, the expression levels of *TOR*, *4EBP*, and *S6K* were not significantly altered (*p* > 0.05, Figure 6B–D). However, additional Leu supplementation led to a decrease in their expression (*p* < 0.05, Figure 6B–D). The expression patterns of *PI3K*, *AKT*, *IRS*, and *ILP* were similar, showing upregulation after *sestrin* silencing and downregulation upon Leu supplementation (*p* < 0.05, Figure 6E,F,H,I). In contrast, *INR* exhibited an opposite expression trend (*p* < 0.05, Figure 6G).

### 3.7. Leu Mediated Antioxidant in Bees Through Sestrin

**Figure 7:**
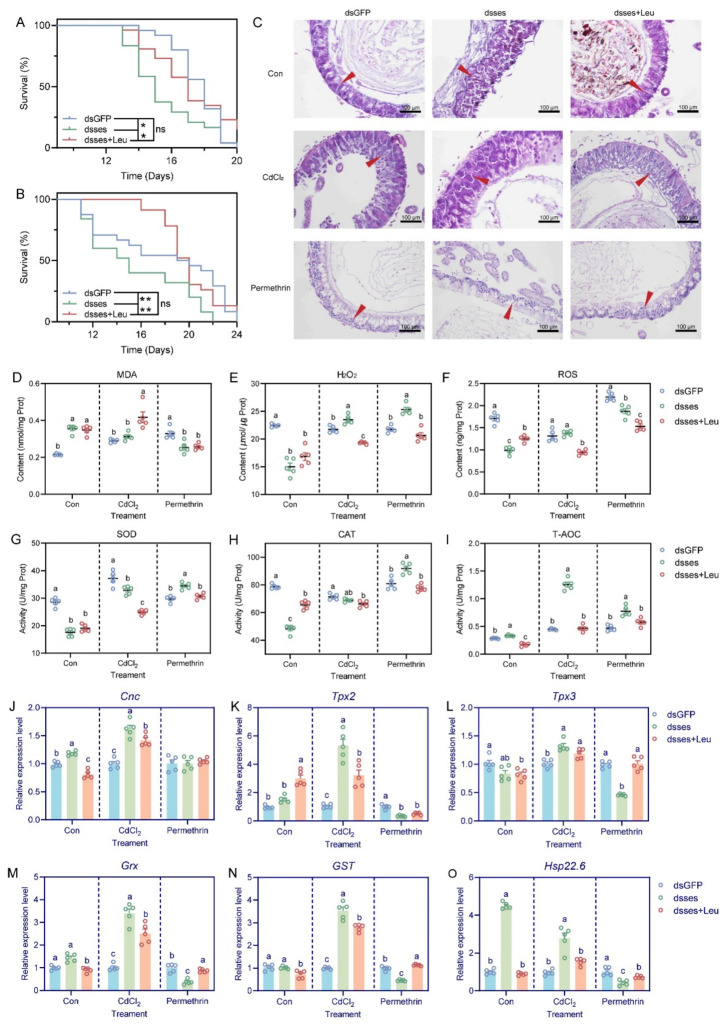
The effect of Leu on the antioxidant function of bees after treatment with dsses. The survival curve of bees exposed to CdCl_2_ (**A**) and permethrin (**B**) after treatment with dsses (*n* = 30). * *p* < 0.05, ** *p* < 0.01. (**C**) The effect of *sestrin* silencing on HE staining of midgut under treatment with sugar, CdCl_2_, and permethrin. The red arrow represents midgut wall. The activity of MDA (**D**), H_2_O_2_ (**E**), and ROS (**F**) after treatment with dsses. The content of SOD (**G**), CAT (**H**), and T-AOC (**I**) after treatment with dsses. The mRNA expression levels of *Cnc* (**J**), *Tpx2* (**K**), *Tpx3* (**L**), *Grx* (**M**), *GST* (**N**), and *Hsp22.6* (**O**) after treatment with dsses (*n* = 5). The data was shown as mean ± SEM. Various letters above the bars indicate significant differences between groups (*p* < 0.05) as determined by one-way ANOVA followed by Tukey’s multiple comparison test.

To further confirm the role of *sestrin* in mediating the antioxidant effects of Leu in bees, we exposed bees to CdCl_2_ or permethrin stress. Exposure to CdCl_2_ or permethrin triggered antioxidant responses in bees, with antioxidants serving as key protective compounds that neutralize free radicals and mitigate oxidative damage to cellular components, including proteins, lipids, and nucleic acids. Survival curve analysis showed that *sestrin* silenced reduced the tolerance of bees to both CdCl_2_ or permethrin, whereas Leu supplementation significantly improved their survival rate (*p* < 0.05, Figure 7A,B). In addition, regardless of whether bees were exposed to CdCl_2_ or permethrin, the dsses group exhibited damage to the midgut intestinal wall compared with the dsGFP group, and this damage was partially alleviated by Leu supplementation (Figure 7C and Figure S2).

We further measured antioxidant markers, antioxidant enzyme activities, and antioxidant-related gene expression following CdCl_2_ or permethrin exposure. The results showed that *sestrin* silenced led to decreased SOD and CAT activities, as well as reduced ROS and H_2_O_2_ levels, whereas Leu supplementation resulted in increased CAT activity and ROS levels (*p* < 0.05, Figure 7E–H). In contrast, T-AOC activity, MDA content, and the transcriptional levels of several antioxidant-related genes (*Cnc*, *Grx*, *Hsp22.6*) were elevated following *sestrin* silenced, and these parameters showed a decreasing trend after Leu supplementation (*p* < 0.05, Figure 7D,I,J,M,O).

When bees were exposed to CdCl_2_ or permethrin, the trends in antioxidant markers, enzyme activities, and gene expression varied. Under CdCl_2_ exposure, *sestrin* silenced resulted in upregulation of T-AOC activity, H_2_O_2_ content, and the expression of antioxidant-related genes (*Cnc*, *Tpx2*, *Grx*, *GST*, *Hsp22.6*), all of which were downregulated following Leu supplementation (*p* < 0.05, Figure 7E,I–O). Under permethrin exposure, *sestrin* silenced led to increased SOD, CAT, and T-AOC activities, as well as elevated H_2_O_2_ content, all of which were decreased after Leu supplementation (*p* < 0.05, Figure 7E,G–I). Furthermore, the transcriptional levels of antioxidant-related genes (*Tpx2*, *Tpx3*, *Grx*, *GST*, *Hsp22.6*) exhibited opposite trends upon *sestrin* silenced and Leu supplementation (*p* < 0.05, Figure 7K–O).

## 4. Discussion

Honeybees play a crucial ecological role in global crop pollination and the maintenance of plant biodiversity [33]. During foraging, workers may encounter various environmental stressors, including pesticides and heavy metals [34]. The primary toxic mechanisms of heavy metals and pesticides are associated with the induction of oxidative stress, accompanied by the generation of ROS [35,36]. ROS can impair the physiological functions of cell membranes by inducing lipid peroxidation (LPO), and MDA is a major end product of this process [37]. When the balance between oxidative stress and antioxidant defense is disrupted, honeybee health may be seriously compromised, which may ultimately impair colony strength and survival [38,39]. To counteract oxidative damage, insects have evolved a complex antioxidant defense system composed of multiple functional proteins and enzymes, including SOD, CAT, thioredoxin peroxidase (Tpx), and glutaredoxin (Grx) [40,41,42]. In addition, T-AOC reflects the overall ability of organisms to resist oxidative damage [43]. In honeybees, H_2_O_2_ serves not only as a signaling molecule but also as an antimicrobial factor to eliminate pathogens [44]. Glutathione S-transferases (GSTs), which are major detoxification enzymes in the antioxidant system, play essential roles in xenobiotic metabolism and in protecting cells against peroxidative damage [45]. Heat shock proteins (HSPs) also participate in the maintenance of redox homeostasis and immune responses [46], and *sHsp22.6* has been reported to possess antioxidant activity [47]. Furthermore, in insects, cap’n’collar (Cnc), the homolog of mammalian Nrf2, is recognized as a central regulator of detoxification enzyme genes and is closely associated with insecticide resistance [48].

Previous studies have shown that enhancing antioxidant defenses through nutritional regulation may help honeybees mitigate the detrimental effects caused by these external stressors. Leu modulated antioxidant status and immunity in juvenile blunt snout bream (*Megalobrama amblycephala*) by regulating antioxidant enzymes, including SOD, Gpx, T-AOC, and CAT, as well as antioxidant-related genes such as *Gpx*, *GST*, and *SOD* [30]. This is consistent with our findings, in which Leu-induced upregulation of TOR is accompanied by increased expression of antioxidant genes and enhanced activities of antioxidant enzymes (CAT and SOD), together with a reduction in ROS levels. However, we noted that Leu supplementation increased MDA levels, which may be attributed to the fact that MDA is stable and less degraded [49]. Thus, the increase in MDA may reflect a homeostatic adjustment of the oxidative response or residual damage from prior oxidative stress. These results suggest that Leu can activate the antioxidant defense system, thereby alleviating the decline in survival caused by CdCl_2_ or permethrin exposure.

As TOR signaling is also considered a key integrative node linking antioxidant capacity and immune function [50], we next investigated how Leu activates the TOR pathway in honeybees. Our results showed that exogenous Leu supplementation modulates TOR activity through upstream components, including *ILP*, *InR*, *PI3K*, *AKT*, and *4EBP*. Notably, Leu increased the transcription levels of these TOR-related genes regardless of whether TOR activity was inhibited, which is consistent with findings in other species and further supports the viewpoint that Leu, as a nutritional signal, effectively activates the TOR signaling pathway through regulation of upstream factors [16].

However, although Leu activated TOR signaling and alleviated oxidative stress, pharmacological inhibition of TOR by rapamycin did not significantly exacerbate stress-induced mortality in honeybees, nor did it markedly alter most antioxidant parameters, including CAT activity, MDA and H_2_O_2_ levels, or the expression of *Cnc*, *Tpx3*, *Grx* and *Hsp22.6*. These findings suggest that, under the experimental conditions of the present study, the TOR pathway may not be the indispensable core pathway through which Leu exerts its antioxidant effects [51]. Although TOR inhibition reduced *Tpx2* expression while increasing T-AOC activity and *GST* expression levels, implying that TOR may participate in the modulation of antioxidant responses, these changes did not appear to have a decisive effect on overall survival. Therefore, we speculate that, in addition to TOR signaling, Leu may regulate antioxidant defense in honeybees through alternative mechanisms, among which a *sestrin*-related pathway is a possible candidate.

Sestrin is considered a key protein that senses intracellular Leu availability and regulates TOR activity in response to nutritional status [52,53]. The sestrin protein sequence in bees is relatively conserved. Research indicated that the N-terminal and C-terminal domains of human sestrin are responsible for scavenging H_2_O_2_-derived radicals and mediating the physical interaction between sestrin and GATOR2, respectively, thereby contributing to the inhibition of mTORC1 activity [54]. Although we predicted the binding sites between leucine and Sestrin in our study, we acknowledge that this prediction lacks experimental validation and therefore requires further verification. Notably, inhibition of TOR signaling with rapamycin did not lead to a reduction in *sestrin* transcription, but instead resulted in a significant increase in its expression. This result suggested that there may be a negative feedback mechanism between TOR and *sestrin* in honeybees. Notably, Leu supplementation restored the rapamycin-induced increase in *sestrin* expression to a normal level, further supporting a negative regulatory interaction between TOR and *sestrin*. On the other hand, silencing *sestrin* by RNAi significantly upregulated the expression of TOR pathway-related genes, including *PI3K*, *AKT*, *IRS*, and *ILP*, whereas Leu supplementation significantly reduced the expression of these genes. Previous studies have shown that *Sesn^−/−^* mice exhibit insulin resistance and enhanced mTORC1 activity [55], while elevated *sestrin* expression under stress conditions suppresses TOR activity [19]. Taken together, these findings suggest that Leu acts as an effective modulator of both TOR signaling and *sestrin* in honeybees, and that reciprocal regulation may exist between them to coordinately integrate metabolic and stress responses. In many cell and tissue types, sestrin promotes autophagy activation through modulation of mTOR activity, which may represent an indirect mechanism by which sestrin alleviates oxidative stress damage [56]. However, the regulatory relationship between TOR and *sestrin* in honeybees remains unclear and requires further investigation.

Beyond its role in sensing nutritional status, *sestrin* is also a stress-inducible protein associated with a variety of stress conditions and is considered a potential antioxidant factor [56]. Under normal physiological conditions, *sestrin* is widely expressed in vivo, and in *Drosophila*, *dsestrin* expression increases with maturation and aging [19]. In the present study, *sestrin* was expressed across all examined tissues of honeybees, with notably high expression at the Pp stage. The prepupal stage (PP-Pb) represents a critical period for tissue remodeling and metamorphosis, during which apoptosis, autophagy, and tissue reorganization occur, processes that are often accompanied by increased oxidative stress [57,58]. As a stress-inducible protein with potential antioxidant functions, the high expression of *sestrin* at the Pp stage may contribute to the elimination of excessive reactive oxygen species generated during metamorphosis, thereby protecting cells from oxidative damage and ensuring successful developmental transition. In addition, *sestrin* functions as a key sensor of nutritional signals, particularly Leu. During the prepupal stage, honeybees undergo substantial changes in nutrient reserves and hormone status. The upregulation of *sestrin* expression at this stage may therefore also be involved in modulating the TOR signaling pathway, thereby coordinating the balance between anabolic and catabolic processes to support proper development.

Previous studies had shown that *sestrin2* knockout exacerbated oxidative stress in mice, as evidenced by reduced SOD activity and increased MDA levels, thereby aggravating cellular and tissue damage [59]. This is generally consistent with our findings. Notably, we also found that the two stressors, CdCl_2_ and permethrin, triggered distinct antioxidant responses. After *sestrin* silencing, honeybees became significantly more sensitive to both stressors. Under CdCl_2_ exposure, SOD activity decreased, whereas the expression of antioxidant-related genes, including *Cnc*, *Grx*, and *Hsp22.6*, increased. In contrast, under permethrin exposure, the activities of SOD, CAT, and T-AOC increased, while the expression of antioxidant genes was downregulated. We speculate that these differences may result from the distinct toxicological mechanisms of the two stressors. CdCl_2_, as a heavy metal, primarily induces oxidative damage by promoting ROS generation and disrupting metallothionein function [60,61]. By contrast, permethrin, as a pyrethroid insecticide, primarily targets sodium channels in the nervous system [62], and oxidative stress is likely a secondary consequence of its toxicity [63]. However, we noted that *sestrin* knockdown led to decreased ROS levels regardless of the presence or absence of exogenous stress, which is inconsistent with previous studies [64]. Therefore, we hypothesized that the effect of *sestrin* on ROS production may be tissue-specific, which needs further investigation. Additionally, *sestrin* knockdown made cells more susceptible to oxidative stress [65], and the resulting severe tissue damage and cell death may lead to metabolic depression in cells, which paradoxically reduces overall ROS production. This could also contribute to the decreased ROS levels observed in our study.

In contrast, Leu exhibited a coordinating and restorative effect on the antioxidant response. Under CdCl_2_ stress, the upregulated expression of antioxidant genes induced by *sestrin* silencing was generally raised following Leu supplementation. Under permethrin stress, the elevated activities of SOD, CAT, and T-AOC caused by *sestrin* silencing were significantly reduced after Leu supplementation. These findings indicated that Leu does not simply enhance or inhibit antioxidant responses in a unidirectional manner through *sestrin*. Rather, in a *sestrin*-dependent context, Leu appears to modulate the antioxidant system toward an appropriate response level according to the specific cellular stress condition. In the absence of *sestrin*, the loss of this important antioxidant regulator led to inadequate antioxidant responses. Nevertheless, exogenous Leu supplementation can partially compensate for the loss of *sestrin* function and re-establish a degree of homeostasis through other, as yet unidentified, pathways. Meanwhile, damage to the intestinal wall caused by CdCl_2_ or permethrin exposure, as well as by *sestrin* silencing itself, may represent an important factor contributing to increased mortality in honeybees [66,67,68]. In addition, Leu may play an important role in preserving the integrity of the honeybee intestinal barrier [69], thereby improving survival under stress conditions.

## 5. Conclusions

This study elucidated the molecular mechanism by which Leu regulates the antioxidant function of honeybees through *sestrin*, and identified *sestrin* as a central hub in this process. The results showed that Leu effectively activates the TOR signaling pathway. However, TOR is not the key mediator of the antioxidant effects of Leu. Furthermore, a complex feedback regulatory relationship exists between *sestrin* and TOR, and sestrin plays an important role in antioxidant protection. Silencing *sestrin* caused structural damage to the honeybee midgut and significantly reduced tolerance to CdCl_2_ or permethrin stress, confirming that *sestrin* is essential for maintaining antioxidant homeostasis in honeybees. Further analyses revealed the functional diversity of *sestrin* in response to different types of oxidative stress: under CdCl_2_ stress, *sestrin* primarily participates in inhibiting excessive activation of antioxidant genes, whereas under permethrin stress, it broadly regulates antioxidant enzyme activities. Under different stress conditions, Leu, acting in coordination with *sestrin*, fine-tunes the overactivated or dysregulated antioxidant system to an appropriate response level. Overall, our findings suggest that Leu integrates nutritional signaling and stress responses through *sestrin* and TOR, thereby contributing to the maintenance of cellular homeostasis in honeybees.

This study provided new insights into the molecular mechanisms by which a nutritional element enhances stress resistance in insects and offered a scientific basis for the development of nutritional intervention strategies for honeybee health management. More importantly, this study identified Leu and *sestrin* as critical regulators of honeybee stress resistance, providing a potential theoretical basis for developing nutritional intervention strategies to improve colony health, reduce stress-related losses, and promote the sustainable development of apiculture.

## Data Availability

The raw data supporting the conclusions of this article will be made available by the authors on request.

## Supplementary Materials

The following supporting information can be downloaded at: https://www.mdpi.com/article/10.3390/biology15141124/s1, Figure S1: Verification of silencing efficiency of dsses; Figure S2: Quantitative histological damage score of honeybee midgut; Table S1: Primer information.

## Abbreviations

The following abbreviations are used in this manuscript:

Leu	Leucine
ROS	Reactive oxygen species
Cd	Cadmium
LD	Target of Rapamycin
RNAi	RNA interference
qRT-PCR	Quantitative real-time PCR
PP	Prepupae
Pw	White-eyed pupae
Pp	Pink-eyed pupae
Pb	Brown-eyed pupae
NE	Newly emerged workers
SOD	Superoxide dismutase
CAT	Catalase
T-AOC	Total antioxidant capacity
MDA	Malondialdehyde
H2O2	Hydrogen peroxide
ANOVA	One-way analysis of variance
LPO	Lipid peroxidation
Tpx	Thioredoxin peroxidase
Grx	Glutaredoxin
GST	Glutathione S-transferases
HSPs	Heat shock proteins
Cnc	Cap’n’collar
