# Supplementary material for: Leucine Enhances Stress Resistance in Honeybees (Apis mellifera L.) by Modulating Sestrin-Dependent Antioxidant Responses
Source: Biology (Basel). 2026 Jul 10;15(14):1124. doi: 10.3390/biology15141124 (PMC13405173; doi:10.3390/biology15141124)
Supplement: Supplementary file 1 [file biology-15-01124-s001.zip › biology-4389292-supplementary.pdf]

Table S1. Primer information

Gene	Primer sequence (5′-3′)	Gene Bank
Primers for gene Silencing		
dsGFP	F: TAATACGACTCACTATAGGGCGAAGTGGAGAGGGTGAAGGTGA<br>R:TAATACGACTCACTATAGGGCGAGGTAAAAGGACAGGGCCATC	XM_006558668.3
dsSestrin	F: TAATACGACTCACTATAGGGCGACCTTACGACTACAGGCAT<br>R: TAATACGACTCACTATAGGGCGACCGCTTCATACGTTCCAT	XM_394521.7
Primers for RT-qPCR		
β-actin	F: CCGTGATTTGACTGACTACCT<br>R: AGTTGCCATTTCCTGTTC	NM_001185145
TOR	F: AAGAATCACCATCACCAGCACTAAG<br>R: AAGTATCGTCCAATTCGGTCCAAC	XM_006566642.3
Sestrin	F: CGCCGAGCATAGTAGGTGAACAG<br>R: TGATCTGGTACGACTCCGACTTCTC	XM_394521.7
4EBP	F: TCAACTACGCCTGGAGGTACTCG<br>R: GCATATTTCGTGGTGGTGTTCGTG	XM_006569699.3
S6K	F: GCATCCTTTCATCGTAGACCTTATG<br>R: CGATAGATAGAAGCAAGCAGTTTCTTC	XM_016911368.2
PI3K	F: ACAACTTCGATTATTAGCGGACAGAG<br>R: GCGTGGGAATTTCAAGAAGACAATG	XM_006570469.3
AKT	F: AACAGTTTCGTGAAAGAGCACATAATG<br>R: TGTCTGGAGTTACAGGAGGAGTTTC	XM_006559055.3
INR	F: GCTCGTGCTCTGCGTCCTC<br>R: GCCTCTCCTGGTTCTTCCTCTTG	XM_026443028.1
IRS	F: ATCGTCCTCTGCTTCCATACATAAAC<br>R: GTGACCAATCCTCCATTATACCATCC	XM_006565041.3
ILP	F: AGTAGTGATCCTAGTGCCAGTAGC<br>R: TGGTGCATTTCTGTCACTGTCTG	XM_026444888.1
Cnc	F:CCAGCCATACACAAGCGGATTC<br>R: CATCTCGGAGAGCCAGCATTTC	XM_006571718.2
Tpx2	F: TGCGACAACCAAGATTCATTACGG<br>R: TGCGTGCATTATCGAGCCATG	XM_016910937.2
Tpx3	F: CCGTAGTGGATGGCGATTTCAAAG<br>R:AACTCAGTAGGGCAGACAAATGTG	NM_001178023.1
Grx	F: GAAAACGCGATGCCTACAAC<br>R: CTGTTCTAGCTCCAGTCATTTCTCC	XM_001123018.5
GST	F: GGACAACCAGTGACACAAGAA<br>R: TGCTCCAATTCACATGCTCCA	XM_624689.5
Hsp22.6	F: CGATGAGCACGGTTGGATTTCAC<br>R: GGTTCTGCTGCTGTTTGGGTG	XM_001119884.5

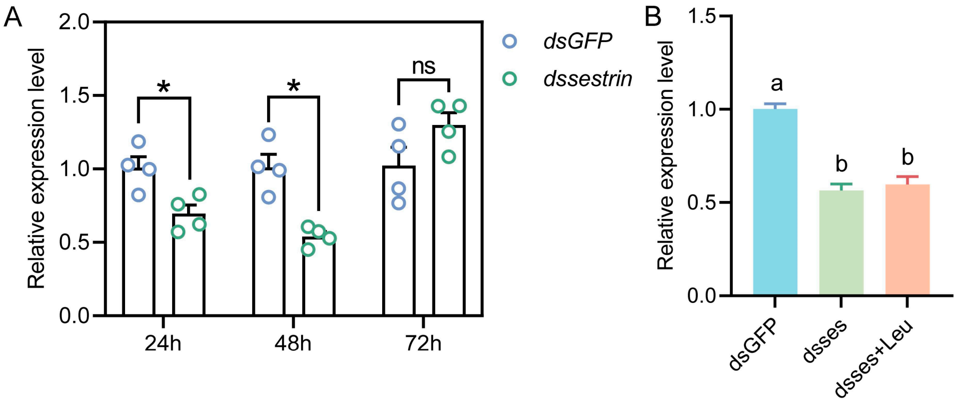

Figure S1. Verification of silencing efficiency of dsses. The expression level of *sestrin* at different time points after a single feeding of dsRNA (A, n=4); B: the expression level of *sestrin* under the experimental conditions with repeated dsRNA treatment (every two days) combined with Leu supplementation (B, n=5). The data was shown as mean ± SEM. The significance of differences between two groups was calculated using a Student's t-test. * $P < 0.05$, ** $P < 0.01$, *** $P < 0.001$. Various letters above the bars indicate significant differences between groups ($P < 0.05$) as determined by one-way ANOVA followed by Tukey's multiple comparison test.

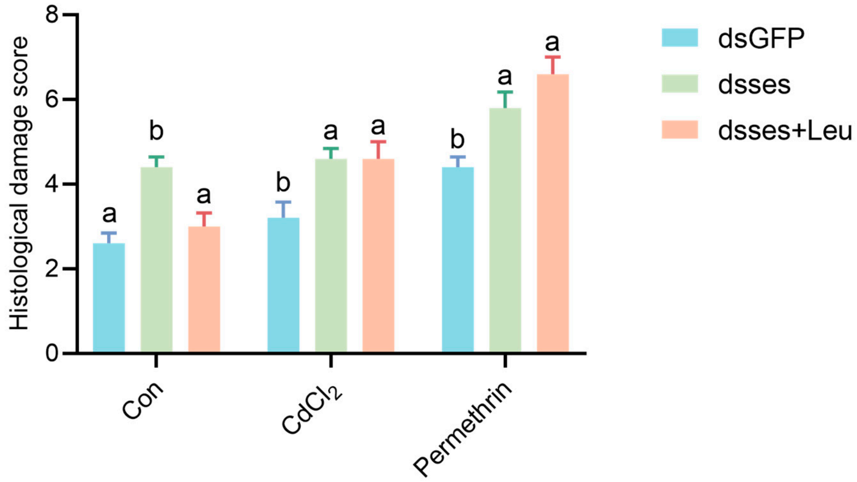

Figure S2. Quantitative histological damage score of honeybee midgut. The score was calculated based on epithelial integrity, and cellular vacuolization (score range: 0-9). Data are presented as means ± SEM from five independent biological replicates. Different letters indicate significant differences among groups ($P < 0.05$, one-way ANOVA with Tukey's post hoc test).
